# Experimentally Induced Empathy Has No Impact on Generosity in a Monetarily Incentivized Dictator Game

**DOI:** 10.3389/fpsyg.2019.00337

**Published:** 2019-03-01

**Authors:** Jan-Erik Lönnqvist, Gari Walkowitz

**Affiliations:** ^1^ Swedish School of Social Science, University of Helsinki, Helsinki, Finland; ^2^ TUM School of Governance, Technical University of Munich, Munich, Germany; ^3^ Center Digitization Bavaria, Munich, Germany; ^4^ Higher School of Economics, National Research University, Moscow, Russia

**Keywords:** empathy, dictator game, generosity, altruism, experimental economics

## Abstract

In a monetarily incentivized Dictator Game, we expected Dictators’ empathy toward the Recipients to cause more pro-social allocations. Empathy was experimentally induced via a commonly used perspective taking task. Dictators (*N* = 474) were instructed to split an endowment of 10€ between themselves and an unknown Recipient. They could split the money 8/2 (8€ for Dictator, 2€ for Recipient) or 5/5 (5€ each). Although the empathy manipulation successfully increased Dictators’ feelings of empathy toward the Recipients, Dictators’ decisions on how to split the money were not affected. We had ample statistical power (above 0.99) to detect a typical social psychology effect (corresponding to *r* around 0.20). Other possible determinants of generosity in the Dictator Game should be investigated.

## Introduction

Behavior that benefits others at a personal cost to the behaving individual is a widespread phenomenon that continues to attract research attention in several fields, including philosophy, evolutionary biology, psychology, and economics. For example, humans invest time and energy in helping other members in their neighborhood (Van Vugt et al., 2000), contribute to charity (Milinski et al., 2002), come to each other’s rescue in crises and disasters (Loewenstein and Small, 2007), and help strangers in spite of potential dangers (Clark and Word, 1974). That human society is abundant with examples of such pro-social behaviors is sometimes considered particularly puzzling among economists, as it challenges the orthodox assumptions of self-interest inherent to many theoretically driven “rational choice” models of human behavior (Camerer, 2003). Nevertheless, a robust body of empirical evidence based on laboratory games – which recreate social interactions in the laboratory using real monetary payoffs – shows that human behavior deviates from economic predictions of profit maximization. The game that best shows this incongruity is the Dictator Game (DG; Kahneman et al., 1986; Forsythe et al., 1994), currently the most prominent paradigm used by economists to investigate why human behavior sometimes deviates from pure self-interest (Bolton et al., 1998; Camerer, 2003; Henrich et al., 2004; Engel, 2011).

The DG is a simple, two player game. Participants are assigned either of two roles: Dictator or Recipient. The Dictator is provided a fixed sum of money, which she splits between herself and the Recipient. Unlike many other games (e.g., the ultimatum game), the Recipient has no power to refuse the money – the game ends after the Dictator’s decision. Giving in the DG is both costly and unconstrained by fear of reprisal or other strategic considerations (Kahneman et al., 1986; Forsythe et al., 1994). Despite this, typical games result in Dictators donating approximately 20–30% of the endowment (Camerer, 2003; Engel, 2011).

Whereas economists have typically sought to explain or model pro-social behaviors with reference to the ultimate causes that could have made such behaviors evolutionarily adaptive, psychologists and recently also more experimentally minded economists have typically focused on the proximate motives that drive individual organisms to engage in those behaviors in the moment (Scott-Phillips et al., 2011). That is, what is it that motivates people to devote their resources to benefitting others? The motivation for all intentional action, including all action intended to benefit others, was long assumed to be egoistic: people were assumed to benefit others because, ultimately, to do so would benefit themselves. The prevailing theories of egoism were challenged by the empathy-altruism hypothesis (Batson and Coke, 1981), according to which empathy, typically defined as “the ability to understand and share in another’s emotional state or context” (Cohen and Strayer, 1996, p. 988), promotes pro-social behavior in ways that cannot be accounted for by self-interest.

The central assertion of the empathy-altruism hypothesis is that empathy evokes altruistic motivation with the ultimate goal of increasing another’s welfare. Note that in contrast to the literature in economics in which altruism is typically defined purely behaviorally as referring to “costly acts that confer economic benefits on other individuals” (Fehr and Fischbacher, 2003, p. 785), psychologists typically refer to altruism as the motivation to increase another person’s welfare (Batson and Powell, 2003). The results from the stream of studies that followed the empathy-altruism hypothesis generally supported the view that empathy causes pro-social behavior. The dispute pertained primarily to whether this process was driven by heightened personal distress caused by the other’s suffering (psychological egoism) or by genuine concern for the other’s well-being (psychological altruism; see Batson and Shaw, 1991).

The empathy-altruism hypothesis suggests that those with higher levels of empathy would be expected to act in a more responsive way to the perceived feelings of another (Batson et al., 1987; Eisenberg and Miller, 1987; Andreoni, 1990; Batson, 1991; Andreoni and Miller, 2002). We therefore expected that increasing Dictator’s empathy toward the Recipient by means of a widely used perspective-taking exercise (Coke et al., 1978; Batson, 1991, 2011; Davis et al., 1996; Stürmer et al., 2005; Ames et al., 2008) would lead the Dictator to make more generous allocations in the DG. Despite the obvious, almost trite, and basically commonsense nature of this hypothesis, we found no research that would directly test it. The closest we came was a study by Batson and Moran (1999), who found it remarkable that although more than 2,000 prisoner’s dilemma studies had been conducted by the early 1990s, none of them had “tested the relatively straight-forward derivation from the empathy-altruism hypothesis that inducing empathy for the other person in a one-trial prisoner’s dilemma will increase cooperation” (p. 911; note that what they denoted co-operation, we would subsume under the more general concept of pro-social behavior). In fact, our hypothesis is even more straightforward than the one tested by Batson and Moran (1999). Although defection in the prisoner’s dilemma is always rational from a self-interested perspective, the ideal outcome in a prisoner’s dilemma is mutual co-operation, providing more complex motives for pro-social behavior than the motives present in the DG (Camerer, 2003; Lönnqvist et al., 2013). Having argued that inducing empathy for the other person in a one-trial prisoner’s dilemma should introduce a new pro-social motive – altruism – and that this motive should increase co-operation, Batson and Moran (1999) found support for this hypothesis. However, this influential study (cited around 300 times) has never been replicated. The small sample size [*N* = 60, i.e., 10 participants in each cell of a 3 (High Empathy vs. Low Empathy vs. Control) × 2 (Business Frame vs. Exchange Frame)] design may raise concerns regarding the replicability of the findings (Open Science Collaboration, 2015). Although there are still many open questions in the current discussion on replicability, there is general agreement that previous social psychology research has often been vastly underpowered and that sample sizes now need to increase. The primary purpose of the present research was thus to investigate, in a large enough sample, to what extent empathy leads DG Dictators to be more generous in their allocations.

Note that Batson and Moran (1999) sought to investigate whether empathy increases altruism. Although allocations in the DG have been described as altruistic (Benenson et al., 2007; Ben-Ner and Kramer, 2011; Israel et al., 2012), we reserve this term to denote the possible psychological motives underlying pro-social behavior. That is, we do not investigate whether empathy leads to altruistic motivation (cf. Batson and Moran, 1999), but whether empathy leads to more generous behavior in the DG, regardless of motives.

## Materials and Methods

### Participants and Procedure

Our study was conducted with 506 participants [mean age = 23.45 (*SD* = 4.03); 59% female] from the University of Cologne (Germany) majoring in different disciplines. Participants were invited via a Cologne Laboratory for Economic Research (CLER) mailing list with approximately 3,700 subscribers who had signed up to take part in experiments. Sixteen experimental sessions were run with 30–32 participants per session. In each session, only two participants were chosen to be Recipients (32 Recipients in all) because we wanted to maximize the number of Dictators given our monetary constraints, leaving us with 474 Dictators (no-one was excluded). Participants did not know that there were only two Recipients in each session. In each session, the two Recipients were randomly matched with two Dictators and paid accordingly.

Upon arrival, participants were randomly (1) seated in computer cubicles that secured anonymity, (2) assigned a role (Dictator or Recipient), (3) paired into dyads, and provided with written instructions. All experimental sessions were conducted on the computer using the experimental platform z-Tree (Fischbacher, 2007). The experiment lasted for about an hour (participants completed a questionnaire after the DG). Subsequently, participants were compensated with a fixed amount of 4€ along with the amount that they earned in the DG.

In the Empathy condition (*N* = 240), Dictators were asked, before making their decision, to write three sentences on a sheet of paper handed out to them about how they imagined the Recipient’s feelings and how their decision would affect the Recipient. They were given 5 min to complete this widely used empathy inducing task (Coke et al., 1978; Fultz et al., 1986; Batson et al., 1988). In the control condition (*N* = 234), participants were asked to write three sentences about yesterday’s weather.

### Measures

At the outset of the DG, the Dictator was provided with an amount of 10€ that was to be distributed between herself and the Recipient. The Dictator could choose the option “8/2” that yielded 8€ for herself and 2€ for the other person or she could choose the option “5/5” that yielded equal payoffs of 5€ for both. The Recipient did not make any decision and the game was played only once. We chose the distribution 8/2 as our selfish option because the mean transfer in DGs is approximately 20–30% of the endowment (Camerer, 2003; Engel, 2011), suggesting that such a division is one that many participants would feel comfortable with [although continuous DGs have become increasingly popular, the game was originally dichotomous (Kahneman et al., 1986) and we wanted to keep it that way for reasons explained below].

As a manipulation check, participants rated on a scale from 0 (*not at all*) to 6 (*very much*) to what extent they felt empathetic, sympathetic, affectionate, warm, compassionate, caring, and concerned in relation to the Recipient. The mean of the seven items was 2.484 (*SD* = 1.343) and Cronbach’s *α* was 0.90. Due to a technical mistake, the results of the manipulation check were not recorded in two of the sessions that we ran, which led to 60 participants having missing data on the manipulation check.

After the manipulation check, participants completed a short questionnaire that contained a measure of personal values and a standard set of demographic variables including sex and age. No other measures were administered. However, as we, at the outset, were interested also in the possible associations between empathy and hypocrisy, the option to flip a coin in order to determine the outcome was included as a parallel experimental manipulation that we report on only briefly. That is, half of the participants received a coin that they could flip in order to decide between the 5/5 and 8/2 outcomes. The coin was placed on the desk of the cubicle in which the Dictators made their decision and the accompanying instructions told Dictators that they could use the coin to determine their choice (it was this aspect of the research design that required us to employ a dichotomous dictator decision). Whether or not the participants were provided with the coin did not affect their decisions (*F* < 1 for both the main effect and the interaction between providing a coin and the empathy manipulation). All results, i.e., manipulation check results and Dictator decisions, were virtually identical for Dictators who received and did not receive a coin.

## Results

The manipulation check showed that the manipulation of empathy was effective. Those primed with empathy scored 2.699 (95% CI: 2.5336–2.865) on feelings of empathy toward the Recipient, whereas those in the control condition scored 2.188 [95% CI: 1.987–2.389; *F* (1, 411) = 15.13, η^2^ = 0.035 (corresponding to an effect size of *d* = 0.39), *p* < 0.001]. Whether or not Dictators received a coin in no way influenced the results of the manipulation check [without coin, those primed with empathy scored 2.654 and those not primed with empathy scored 2.157 (*d* = 0.35, *p* < 0.05); with coin, those primed with empathy scored 2.744 and those not primed with empathy scored 2.204 (*d* = 0.44, *p* < 0.001)]. All analyses were run with sex, age, and their interactions with other variables as covariates (including the abovementioned parallel manipulation with/without coin), but as the results were always virtually identical, only analyses without covariates are presented.

**Figure 1:**
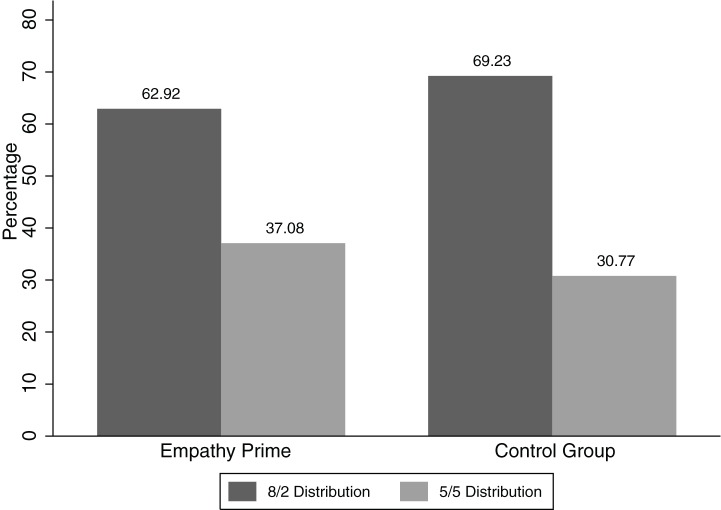
Frequency of 8/2 and 5/5 choices in the empathy and in the control condition.

Among those 240 Dictators who received the empathy prime, 151 (62.92%) chose the 8/2 and 89 (37.08%) chose the 5/5 distribution. Among the 234 Dictators in the control condition, 162 (69.23%) chose the 8/2 and 72 (30.77%) chose the 5/5 distribution (see Figure 1). A chi-square test of independence was calculated comparing the frequency of the 5/5 choice among those primed with empathy and those in the control group. The difference between the two conditions (empathy vs. control) was not statistically significant [χ^2^(1) = 2.106, ω = 0.067 (95% CI: −0.024–0.154), *p* = 0.147]; this difference was also not significant when looking separately at Dictators who did not receive a coin and Dictators who did receive a coin [χ^2^(1) = 0.4554 and χ^2^(1) = 0.1970, respectively, both *p* > 0.15]. The effect size ω = 0.067 corresponds directly to an effect size of *r* = 0.067. Sensitivity analyses showed that we would have had an above 99% chance of detecting a typical social psychology effect size (*r* = 0.20; Richard et al., 2003; Stanley et al., 2019).

## Discussion

The current experiment tested the hypothesis that increasing Dictator’s empathy toward the Recipient would lead the Dictator to make more generous allocations in the DG. This hypothesis was refuted by the empirical evidence. We were thus with an eight-fold number of participants and a more straightforward experimental setup – the DG is motivationally less complex than the prisoner’s dilemma – unable to reproduce the positive significant effect of empathy on pro-social behavior reported by Batson and Moran (1999). Importantly, the experimental manipulation of empathy was successful: Dictators instructed to imagine the Recipient’s feelings felt more empathy toward the Recipient. There were also no ceiling or roof effects that could have distorted the results – in all, 161 (33.97%) of our 474 Dictators chose the equal allocation.

Although there is no one-to-one correspondence between pro-social behavior and altruism and we did not set out to investigate whether empathy leads to altruistic motivation, our results do speak to the empathy-altruism hypothesis. Because empathy did not increase pro-social behavior, it seems reasonable to conclude that it did not cause altruistic motivation, or at least not strong altruistic motivation. This can be considered surprising, taking into account that the empathy-altruism hypothesis has been supported by a large number of social psychology studies. One important advantage of investigating pro-social behavior in a monetarily incentivized DG is that it requires individuals to put their money where their mouth is. This is typically not the case in social psychology experiments. It seems possible that the altruistic motivation caused by empathy is so weak that even moderate monetary incentives (recall that our Dictators chose between an 8/2 and 5/5 allocation of 10€) will override it. In case the stakes had been smaller (or hypothetical), Dictators could have been more inclined to act upon their feelings of empathy. Empathy has indeed been suggested to increase only superficial minimal-cost helping (Neuberg et al., 1997). Consistent with this line of thought, the study by Batson and Moran (1999) that found a positive effect for empathy required participants to allocate lottery tickets, and the mean expected pay-off was only $0.50. Employing at least moderate monetary incentives in future studies on the effects of empathy on pros-social behavior and altruistic motivation could be highly revealing.

Some explanations of DG behavior allude to morality. People generally wish to consider themselves moral (Aquino and Reed, 2002), and judgments about right and wrong could thus guide Dictators’ behavior. Some researchers within moral psychology have viewed empathy as a source of principled moral judgment (Nussbaum, 1996, 2001; Haidt, 2003) and as a mechanism selected by evolution as a proximate mechanism to generate altruism (De Waal, 2008). If an equal allocation (5/5) is judged more moral than an unfair allocation (8/2), our results suggest that feeling empathy toward someone does not always lead to more moral behavior toward that person. However, we also note that even though the manipulation of empathy was effective, empathy was still rather low, well below the midpoint of the empathy scale. One reason that empathy has by some researchers been argued to be a poor guide to moral judgments is that we may feel empathy only toward in-group members (Batson, 2011; Jordan et al., 2016; Bloom, 2017). It seems possible that the anonymous Recipient was not considered an in-group member and thus failed to elicit much empathy. The interplay or even interdependence of empathy and group membership (in-group vs. out-group Recipient) in the determination of Dictator behavior should be an interesting topic for future research.

Some other limitations of the present research need to be acknowledged. The independent variable, the manipulation check, and the dependent variable can all be criticized. Our manipulation was rather broad in the sense that it did not allow us to differentiate between affective and cognitive components of empathy. Regarding the manipulation check, the items that we employed to assess the effectiveness of the manipulation were very blatant in their content and could have been subject to demand effects – participants in the empathy condition could have noticed the connection between the instructions to write about “how they imagined the Recipients’ feelings and how their decision would affect the Recipient” and the items asking them to rate how much they felt “sympathetic”, “caring”, etc. A standardized questionnaire allowing for the assessment of various components of empathy would have been preferable as a manipulation check. It is now possible that participants did not really feel empathy but were simply providing answers they thought the experimenter wanted. These lines of criticism all suggest that the manipulation was at fault – it did not really induce empathy (only demand effects), it did not induce the relevant type of empathy, or it induced only weak empathy. These are all valid criticisms, all the more important because we employed the most commonly used and face-valid manipulation of empathy that we could find. How exactly empathy and other states can best be manipulated and how the success of such manipulations can best be measured are very basic and very important topics for future research. Regarding the dependent variable, constraining the action space of the Dictator to a dichotomous choice could be argued to have affected the behavior of the Dictator. However, meta-analyses that have investigated the effects of action space on Dictator behavior have, depending on the type of analysis, found no statistically significant effects or extremely small effects (amount of explained variance < 0.001) for action space on Dictator behavior (Engel, 2011). Nevertheless, in the absence of prior empirical research directly comparing dichotomous and more continuous measures of DG behavior, we are forced to acknowledge that a more continuous measure of DG behavior could have given a different result. A continuous measure of DG giving would also have given us slightly more power to detect a small effect.

Our results could be sensitive to population differences. For example, non-student populations could be more affected by empathy (students of at least some disciplines, such as philosophy, may be less affected by feelings when making moral judgments; Haidt, 2001). Nevertheless, we caution that moderator effects are best detected when the relation between predictor and outcome is substantial (Chaplin, 1991). The present results show that empathy is even at best not a strong predictor of DG giving, implying that statistical tests of interaction effects would require extremely large samples.

We are also forced to acknowledge that our sample size was not only too small to investigate possible moderator effects, but it was also too small to rule out the possibility of a true small effect. Although we had a 99% chance of detecting a typical social psychology effect (*r* = 0.20), we would have needed 1,076 participants – above double the amount that we actually had – to have 80% power to detect a small effect (*r* = 0.10). The reason for the lack of prior published research on the hypothesis that we set out to test; i.e., that empathy would lead the Dictator to make more generous allocations in the DG, could be that the effect is so small that the results of prior empirical studies have typically not reached conventional levels of statistical significance. Publication bias could have led to under-reporting of such findings. In fact, chronically low statistical power and some degree of selective reporting bias have led to an overestimation of effect sizes in social psychology (Stanley et al., 2019), which means that if there is a small effect of empathy of DG giving, something we cannot rule out with the current sample size, it could well be that the size of that effect is not that different from the real size of a typical social psychology effect.

Also supporting the view that empathy, even at best, is not a strong predictor of DG behavior is that research on the effects of trait empathy on DG giving has produced miscellaneous results. A currently popular view in research on individual differences is that states can be viewed as density distributions of states (Fleeson, 2001). This would suggest that one could expect similar behavioral effects for traits and states. Research on the effects of trait empathy on DG giving has produced mixed results, with some studies finding an effect for some aspects of empathy (Edele et al., 2013) and some not finding an effect (Artinger et al., 2014; Zhao et al., 2017). These results together with our results suggest that if an effect does exist, it is likely to be rather small.

Sample size is more generally an issue within research on the DG. Many of the most-cited papers, with the number of citations running into the thousands, could be underpowered, with sample sizes beginning from the 20s and rarely reaching even 200 participants, even when collapsing data across all sub-studies, conditions, or treatments (e.g., Eckel and Grossman, 1996, 1998; Hoffman et al., 1996; Haley and Fessler, 2005). The hazards of small sample sizes are well illustrated by the entire research programs on the “watching eyes” or “minimal social cues” phenomena that were inspired by one study with 124 dictators (Haley and Fessler, 2005). This frenzy of research resulted in numerous high-impact publications from different laboratories. But more recent large-scale studies (Raihani and Bshary, 2012) and meta-analysis (Northover et al., 2017) have cast much doubt on the existence of the phenomena.

The above referred to research on “watching eyes” is only one small example of the literature that has sought the determinants of pro-social behavior in the DG. Highly cited researches conducted according to the stringent scientific procedures set out by the experimental economics community (e.g., real monetary incentives, no deception) have identified for instance guilt (Gummerum et al., 2010), inequality aversion (Fehr and Schmidt, 1999), moral costs (Brañas-Garza, 2007), and affect (Schulz et al., 2014) as determinants of pro-social behavior in the DG (the sample sizes of these studies vary from the low 20s to just under 140). The designs and conditions in which the experiments have been run could be argued to be rigorous and free of bias, making the general lack of statistical power considerations all the more surprising. This raises the possibility that many of these studies, and perhaps most other studies, on the determinants of DG are likely to have been underpowered. Feelings of empathy, although not a determinant of DG giving according to our results, could be expected to be strongly associated with many of the above suggested determinants of DG behavior, such as guilt (e.g., Leith and Baumeister, 1998). We therefore wish to raise the question of the replicability of previous findings and suggest that the determinants of DG behavior need to be explored further. It could be that we actually know very little about what drives behavior in the DG and thus also very little about what the DG actually measures.

## Data Availability

The datasets generated for this study can be found in the [Open Science Framework] doi: 10.17605/OSF.IO/HFD6W.

## Ethics Statement

The authors declare that full review and approval by an ethics committee were not required according to the local guidelines. However, experiments run at the Cologne Laboratory for Economic Research must conform to the guidelines of the experimental economics society. Written informed consent was obtained from all participants.

## Author Contributions

J-EL and GW conceived, designed, and performed the experiments; analyzed the data; and wrote the paper.

### Conflict of Interest Statement

The authors declare that the research was conducted in the absence of any commercial or financial relationships that could be construed as a potential conflict of interest.
